# Approaches for Detection of Hepatitis B Virus Pre-S Gene Deletions and Pre-S Deleted Proteins and Their Application in Prediction of Higher Risk of Hepatocellular Carcinoma Development and Recurrence

**DOI:** 10.3390/v14020428

**Published:** 2022-02-18

**Authors:** Yueh-Te Lin, Long-Bin Jeng, Ih-Jen Su, Chiao-Fang Teng

**Affiliations:** 1 Cancer Genome Research Center, Chang Gung Memorial Hospital, Taoyuan 333, Taiwan; dawnbread1207@gmail.com; 2 Organ Transplantation Center, China Medical University Hospital, Taichung 404, Taiwan; longbin.cmuh@gmail.com; 3 Department of Biotechnology, Southern Taiwan University of Science and Technology, Tainan 710, Taiwan; suihjen0704@stust.edu.tw; 4 Graduate Institute of Biomedical Sciences, China Medical University, Taichung 404, Taiwan; 5 Research Center for Cancer Biology, China Medical University, Taichung 404, Taiwan

**Keywords:** hepatocellular carcinoma, hepatitis B virus, pre-S deleted proteins, pre-S gene deletions, detection approaches

## Abstract

Hepatocellular carcinoma (HCC) is among the most common and lethal human cancers worldwide and is closely associated with chronic hepatitis B virus (HBV) infection. Pre-S deleted proteins are naturally occurring mutant forms of HBV large surface proteins that are expressed by HBV surface genes harboring deletion mutations over the pre-S gene segments. It has been well demonstrated that HBV pre-S deleted proteins function as important oncoproteins, which promote malignant phenotypes of hepatocytes through the activation of multiple oncogenic signaling pathways and result in HCC formation. The oncogenic signaling pathways activated by pre-S deleted proteins have been verified as potential therapeutic targets for the prevention of HCC development. Moreover, the presence of pre-S gene deletions and the expression of pre-S deleted proteins in the blood and liver tissues of HBV-infected patients have been evaluated as valuable biomarkers for predicting a higher risk of HCC development and recurrence after curative surgical resection. Therefore, the precise detection of pre-S gene deletions and pre-S deleted proteins holds great promise as regards identifying the patients at higher risk of HCC development and recurrence, thus aiding in more timely and better treatments to improve their survival. This review summarizes the major approaches used for the detection of pre-S gene deletions and pre-S deleted proteins, including the approaches based on Sanger DNA sequencing, pre-S gene chips, next-generation sequencing and immunohistochemistry staining, and it highlights their important applications in the prediction of higher risks of HCC development and recurrence.

## 1. Introduction

As a predominant form of primary liver cancer, hepatocellular carcinoma (HCC) accounts for nearly 90% of all liver malignancies [1,2]. Despite significant advances made in detection, prevention, and therapy, HCC is still the six most frequently diagnosed human cancer and the third leading cause of cancer-related death worldwide, leading to approximately 800,000 deaths annually [3,4]. Although curative surgical resection is available for HCC patients, the recurrence rate of HCC within five years after surgery is as high as 70%, leading to poor patient outcomes [5,6]. Therefore, the development of valuable biomarkers, along with approaches for the precise detection of the biomarkers, is still a key goal for the early identification and timely treatment of patients at higher risk of HCC development and recurrence, thus helping to improve their survival.

Chronic hepatitis B virus (HBV) infection is an important causative factor for HCC development and accounts for more than 50% of HCC cases worldwide [7,8]. The HBV surface gene is composed of three successive gene segments (pre-S1, pre-S2, and S) and is responsible for the expression of three different sizes of surface proteins (small, middle, and large) from the S segment, the pre-S2 and S segments, and all three gene segments, respectively [9,10]. Several deletion mutations that naturally occur in either or both of the pre-S1 and pre-S2 gene segments (collectively called pre-S gene deletions) have been identified and result in the expression of mutant forms of large surface proteins (called pre-S deleted proteins) [11,12]. Pre-S deleted proteins have been demonstrated to work as important HBV oncoproteins that induce HCC development through the activation of multiple oncogenic signaling pathways to trigger hepatocyte malignant transformation [13,14,15]. The inhibition of the oncogenic signaling pathways activated by pre-S deleted proteins has shown potential efficacy in the prevention of HCC development [16,17,18]. Moreover, patients infected with HBV with the presence of pre-S gene deletions and the expression of pre-S deleted proteins in the blood and liver tissues have been associated with a higher risk of HCC development and recurrence after curative surgical resection than patients without HBV deletion mutants [19,20]. Therefore, pre-S gene deletions and pre-S deleted proteins are valuable biomarkers of HCC development and recurrence; the precise detection of their presence and expression in clinical specimens is important in identifying high-risk patient populations for better management and outcome improvement.

**Figure 1:**
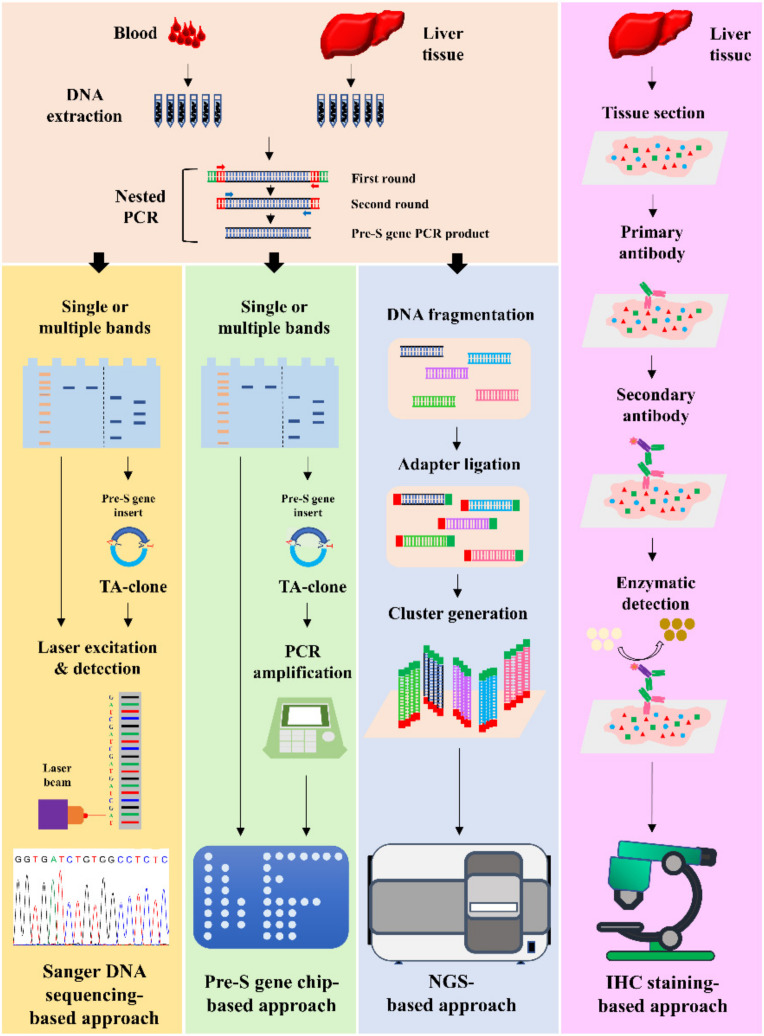
Schematic summary of four major approaches to the detection of HBV pre-S gene deletions and pre-S deleted proteins. The pre-S gene deletions are detected by approaches based on Sanger DNA sequencing, pre-S gene chips, and NGS. For the Sanger DNA sequencing-based approach, the pre-S gene PCR products are prepared from the blood or liver tissues by a nested PCR and then resolved on agarose gels. In cases where only a single PCR band is visualized, the pre-S gene PCR products are directly analyzed by Sanger DNA sequencing. In cases where multiple PCR bands are visualized, the individual PCR bands are separately directed to TA cloning, followed by Sanger DNA sequencing. Through the detection of the sequences, which are deleted in the pre-S gene segments, the presence and type of pre-S gene deletions can be determined. For the pre-S gene chip-based approach, the pre-S gene PCR products are also prepared from the blood or liver tissues by a nested PCR and resolved on agarose gels. In cases where only a single PCR band is visualized, the pre-S gene PCR products are directly subjected to chip hybridization. In cases where multiple PCR bands are visualized, the individual PCR bands are first separately directed to TA cloning, followed by PCR-based amplification of the pre-S gene inserts in multiple clones for chip hybridization. Through the detection of the signals, which are negative on probes in the chip, the presence and type of pre-S gene deletions can be determined. For the NGS-based approach, the pre-S gene PCR products prepared from the blood or liver tissues are directly subjected to NGS analysis without the need for agarose gel electrophoresis and regardless of single or multiple PCR bands. Through the detection of the frequency of deleted pre-S gene DNA fragments, the presence, type, and percentage of pre-S gene deletions can be determined. In addition, the pre-S deleted proteins are detected by an IHC staining-based approach, in which the expression of HBV surface proteins in the liver tissues is detected by IHC staining for GGH visualization. Via the detection of the type of GGH, the presence and type of pre-S deleted proteins can be determined. Abbreviations: HBV, hepatitis B virus; PCR, polymerase chain reaction; NGS, next-generation sequencing; TA, thymine–adenine; IHC, immunohistochemistry; GGH, ground glass hepatocyte.

Four major approaches have been used to detect pre-S gene deletions and pre-S deleted proteins in the blood and liver tissues, including approaches based on Sanger DNA sequencing, pre-S gene chips, next-generation sequencing (NGS), and immunohistochemistry (IHC) staining. This review summarizes the methodology and applications of these approaches in the detection of pre-S gene deletions and pre-S deleted proteins as biomarkers for predicting a higher risk of HCC development and recurrence (Figure 1 and Table 1).

**Table 1:** Summary of four major approaches for the detection of HBV pre-S gene deletions and pre-S deleted proteins and their applications in the prediction of a higher risk of HCC development and recurrence.

I. Sanger DNA Sequencing-Based Approach				
References	Sample Source	Detection Method	Detection Target	Clinical Application
[21,22]	Serum	Sanger DNA sequencing	Pre-S gene deletions	Presence of either or both of pre-S1 and pre-S2 gene deletions as an independent biomarker for higher risk of HCC development
[23]	Serum	Sanger DNA sequencing	Pre-S gene deletions	Presence of pre-S2 gene deletions between nts 38 and 55 as an independent biomarker for higher risk of HCC development
[24]	Serum	Sanger DNA sequencing	Pre-S gene deletions	Presence of pre-S gene deletions, especially pre-S2 gene deletions, as an independent biomarker for higher risk of HCC recurrence after curative surgical resection
**II. Pre-S Gene Chip-Based Approach**				
References	Sample source	Detection method	Detection target	Clinical application
[25]	Serum	Pre-S gene chip	Pre-S gene deletions	High percentage of pre-S2 gene deletions (≥5% of clones) as an independent biomarker for higher risk of HCC recurrence after curative surgical resection
**III. NGS-Based Approach**				
References	Sample source	Detection method	Detection target	Clinical application
[26]	Plasma	NGS	Pre-S gene deletions	Either the presence of deletions spanning the pre-S2 gene segment or high percentage of pre-S2 plus pre-S1 + pre-S2 gene deletions (>25% of pre-S gene DNA fragments), or a combination of both factors, as an independent biomarker for higher risk of HCC recurrence after curative surgical resection
[27]	Plasma	NGS	Pre-S gene deletions	Presence of pre-S2 gene deletions at nts 1 to 54 as an independent biomarker for higher risk of HCC recurrence after curative surgical resection
**IV. IHC Staining-Based Approach**				
References	Sample source	Detection method	Detection target	Clinical application
[28,29]	Liver tissues	IHC staining	Pre-S deleted proteins	High expression score of type II GGHs (pre-S2 deleted proteins; ≥10% of hepatocytes) as an independent biomarker for higher risk of HCC recurrence, no matter whether the subject is receiving pre-surgical anti-HBV treatment

Abbreviations: HBV, hepatitis B virus; HCC, hepatocellular carcinoma; NGS, next-generation sequencing; nts, nucleotides; IHC, immunohistochemistry; GGHs, ground glass hepatocytes.

## 2. Sanger DNA Sequencing-Based Detection of Pre-S Gene Deletions and Its Application in Prediction of Higher Risk of HCC Development and Recurrence

The Sanger DNA sequencing-based approach aims to amplify the entire pre-S1 and pre-S2 gene segments via a nested polymerase chain reaction (PCR) assay, followed by Sanger DNA sequencing to detect the presence and type of deletion mutations over the pre-S gene segments [30]. In the first step, DNA samples are extracted from the blood or liver tissues and used as a template for the amplification of the pre-S gene segments via two successive rounds of PCR (called nested PCR). In the second step, the pre-S gene PCR products are resolved by agarose gel electrophoresis. In cases where only a single PCR band is visualized, the pre-S gene PCR products are directly analyzed by Sanger DNA sequencing. In cases where two or more different sizes of PCR bands are visualized, the third step is needed, in which the individual PCR bands on agarose gels are separately excised, followed by thymine–adenine (TA) cloning, and the resulting pre-S gene insert-containing clones are subjected to Sanger DNA sequencing analysis.

By using the Sanger DNA sequencing-based approach, Chen et al. detected the presence and type of pre-S gene deletions in the serum of 141 chronic hepatitis B patients without HCC, and, in a follow-up period of 10 years, showed that patients infected with HBV with either or both pre-S1 and pre-S2 gene deletions had a five-fold higher risk of HCC development than patients without HBV deletion mutants [21]. Sinn et al. used the Sanger DNA sequencing-based approach to analyze another cohort of 195 chronic hepatitis B patients and observed a similar result showing that the five-year cumulative incidence rate of HCC development in patients infected with HBV with pre-S gene deletions was five-fold higher than patients without HBV deletion mutants [22]. The studies conducted by Chen et al. and Sinn et al. consistently revealed that the presence of pre-S gene deletions in the serum serves as a biomarker that independently predicts a higher risk of HCC development [21,22]. Furthermore, Cohen et al. analyzed 302 chronic hepatitis B patients and showed that the Sanger DNA sequencing-based detection of pre-S2 gene deletions between nucleotides (nts) 38 and 55 (a 12 nt, 15 nt, or 18 nt deletion) in the serum independently predicted a higher risk of HCC development [31]. In addition, Li-Shuai et al. analyzed pre-S gene deletions in the serum of 113 HBV-related HCC patients who received curative surgical resection by using the Sanger DNA sequencing-based approach and showed that patients infected with HBV with pre-S gene deletions, especially pre-S2 gene deletions, had a higher risk of HCC recurrence than patients without HBV deletion mutants after surgery [32].

## 3. Pre-S Gene Chip-Based Detection of Pre-S Gene Deletions and Its Application in Prediction of Higher Risk of HCC Recurrence

The pre-S gene chip-based approach aims to amplify the entire pre-S1 and pre-S2 gene segments via a nested PCR assay, followed by hybridization with the pre-S gene chip to detect the presence and type of deletion mutations over the pre-S gene segments [33]. A pre-S gene chip is comprised of 42 oligonucleotide DNA probes, which together cover the pre-S gene segments [33]. In the first few steps, similar to the Sanger DNA sequencing-based approach, the pre-S gene PCR products from the blood or liver tissues are prepared via a nested PCR assay and resolved on agarose gels. In cases where only a single PCR band is visualized, the pre-S gene PCR products are directly subjected to chip hybridization. In cases where multiple PCR bands are visualized, the pre-S gene PCR products are first directed to TA cloning, followed by the PCR-based amplification of the pre-S gene inserts in multiple clones, and the resulting PCR products are analyzed with the pre-S gene chip. Through the detection of the signals, which are negative on probes in the chip, the presence and type of pre-S gene deletions can be determined.

By using the pre-S gene chip-based approach, Yen et al. analyzed the presence and type of pre-S gene deletions in the serum of 175 HBV-related HCC patients receiving curative surgical resection, in which 30 independent pre-S gene insert-containing clones from each serum were analyzed for the semiquantitative detection of pre-S gene deletions [25]. Their results show that the percentage of overall pre-S gene deletions, especially pre-S2 gene deletions, was higher in patients with HCC recurrence than in patients without after surgery [25]. Patients infected with HBV with a high percentage of pre-S2 gene deletions (≥5% of clones) had a higher risk of HCC recurrence than patients with a low percentage of pre-S2 gene deletions [25]. The high percentage of pre-S2 gene deletions in the serum was determined as an independent biomarker for predicting a higher risk of HCC recurrence after curative surgical resection [25].

## 4. NGS-Based Detection of Pre-S Gene Deletions and Its Application in the Prediction of Higher Risk of HCC Recurrence

The NGS-based approach aims to amplify the entire pre-S1 and pre-S2 gene segments via a nested PCR assay, followed by NGS analysis to detect the presence, type, and percentage of deletion mutations over the pre-S gene segments [34]. Different from Sanger DNA sequencing, NGS carries out massive parallel sequencing, during which millions of DNA fragments from a single sample are sequenced in unison [35,36]. Similar to the Sanger DNA sequencing-based and pre-S gene chip-based approaches, in the first step, a nested PCR assay is performed to prepare the pre-S gene PCR products from the blood or liver tissues. However, unlike these approaches, the pre-S gene PCR products do not need to be resolved by agarose gel electrophoresis and directed to TA cloning; instead, the PCR products, regardless of whether they contain single or multiple bands, are directly subjected to NGS analysis for both qualitative and quantitative detection of pre-S gene deletions.

Using the NGS-based approach, Teng et al. detected the presence, type, and percentage of pre-S gene deletions in the plasma of 75 HBV-related HCC patients receiving curative surgical resection, and showed that patients infected with HBV with either the presence of deletions spanning the pre-S2 gene segment or a high percentage of pre-S2 plus pre-S1 + pre-S2 gene deletions (>25% of pre-S gene DNA fragments), or a combination of both factors, displayed a higher risk of HCC recurrence than patients without HBV deletion mutants after surgery [26]. Either the presence of deletions spanning the pre-S2 gene segment or a high percentage of pre-S2 plus pre-S1 + pre-S2 gene deletions (or both) were evaluated as an independent biomarker for predicting a higher risk of HCC recurrence [26]. Furthermore, Teng et al. used the same approach to determine that the pre-S2 gene deletions at nts 1 to 54 (a 54 nt deletion) were the most frequently detected type of pre-S gene deletions in the plasma, and are a biomarker that independently predicts a higher risk of HCC recurrence after curative surgical resection [27].

## 5. IHC Staining-Based Detection of Pre-S Deleted Proteins and Its Application in Prediction of Higher Risk of HCC Recurrence

The IHC staining-based approach aims to detect ground glass hepatocytes (GGHs) by the IHC staining of HBV surface proteins, followed by the quantification of GGHs with a semiquantitative expression scoring system [28]. Two distinct types of GGHs (type I and type II) have been identified in liver tissues and express specific types of pre-S deleted proteins with different patterns of intracellular distribution [11,12]. The type I GGHs grow in scatters and express an inclusion-like pattern of large surface proteins that harbor pre-S1 gene deletions (called pre-S1 deleted proteins), whereas the type II GGHs grow in clusters and express a peripheral pattern of large surface proteins that harbor pre-S2 gene deletions (called pre-S2 deleted proteins) [11,12]. Because of these characteristics of GGHs, the detection of the type and expression score of GGHs can determine the type and expression level of pre-S deleted proteins in the liver tissues.

By using the IHC-based approach, Tsai et al. detected the type and expression score of GGHs in the liver tissues of 82 HBV-related HCC patients receiving curative surgical resection, and showed that patients infected with HBV with a high expression score of type II GGHs (≥10% of hepatocytes) had a higher risk of HCC recurrence than patients with low expression scores of type II GGHs after surgery [28]. The high expression score of type II GGHs in the liver tissues was evaluated as an independent biomarker for predicting a higher risk of HCC recurrence, especially for late local recurrence (more than one year after surgery) [28]. Furthermore, Tsai et al. used the same approach to analyze another cohort of 186 HBV-related HCC patients who underwent curative surgical resection, among whom 82 patients received anti-HBV therapy before the surgery, and showed that the high expression score of type II GGHs independently predicted a higher risk of HCC recurrence, regardless of whether patients received pre-surgical anti-HBV treatment [29]. Collectively, these results validate a clinical correlation between the expression of pre-S2 deleted proteins in the liver tissues and HCC recurrence after curative surgical resection.

## 6. Discussion

On the basis of their different methodologies, the four major approaches introduced in this review have different advantages and disadvantages in terms of the detection of pre-S gene deletions and pre-S deleted proteins (Table 2). The Sanger DNA sequencing-based approach is practically easy to operate and provides sequence information; however, the process of agarose gel electrophoresis and TA cloning is somewhat time consuming and involves high risks of omitting bands with too-low intensities or too-similar sizes in agarose gels [34]. The pre-S gene chip-based approach is a little more time efficient than the Sanger DNA sequencing-based approach due to the use of a gene chip instead of Sanger DNA sequencing; however, this approach has the same disadvantages as the Sanger DNA sequencing-based approach and cannot provide sequence information [33]. The NGS-based approach is much more efficient, sensitive, and accurate than the Sanger DNA sequencing-based and pre-S gene chip-based approaches, because this approach does not need to perform agarose gel electrophoresis and TA cloning, and can provide not only qualitative, but also quantitative, results for the detection of pre-S gene deletions; however, this approach requires support from skilled instrument technicians and bioinformatics analysts and is more expensive [34]. The IHC staining-based approach is the only one of these approaches to detect the expression pattern of pre-S deleted proteins in the liver tissues; however, because the liver tissue sections used for IHC staining were partial liver tissues, the results obtained by this approach may insufficiently represent the whole liver tissue [37]. The NGS-based detection of pre-S gene deletions in plasma has been shown to determine the patterns of pre-S deleted proteins in liver tissues more efficiently and accurately than IHC staining-based detection [37]. Moreover, unlike DNA samples, which can be amplified by PCR assays, the detection of protein expression may be limited by proteins present at levels that are too low. Therefore, when detecting pre-S gene deletions and pre-S deleted proteins as biomarkers for predicting a higher risk of HCC development and recurrence, it is recommended to take into consideration the advantages and disadvantages of the approaches used so as to improve the prediction fidelity.

Many cellular and animal studies have effectively demonstrated that pre-S deleted proteins can promote hepatocyte malignant transformation to induce HCC development through the activation of multiple oncogenic signaling pathways, and the inhibition of the oncogenic signaling pathways activated by pre-S deleted proteins has a preventive effect on HCC development [15,16,17,18,20]. However, the expression profiles of the pre-S deleted protein-activated oncogenic signaling pathways in the liver tissues of patients infected with HBV with pre-S gene deletions remain largely unclarified. Recently, Teng et al. showed that HCC patients infected with HBV, with either the presence of deletions spanning the pre-S2 gene segment or a high percentage of pre-S2 plus pre-S1 + pre-S2 gene deletions, displayed higher numbers and activity levels of regulatory T cells (Tregs) and higher expression levels of immune checkpoint programmed death ligand 1 (PD-L1) in tumor tissues than patients without HBV deletion mutants [38,39]. Because the increased infiltration and activation of Tregs and the increased expression of PD-L1 in HCC tissues have been associated with poor prognoses [23,24,40], our results suggest that inhibitors targeting Tregs and PD-L1 are promising therapeutics for treating HCC patients infected with HBV with pre-S gene deletions. The identification of patients at higher risk of HCC development and recurrence via the detection of pre-S gene deletions and pre-S deleted proteins, followed by the investigation of the expression profiles of oncogenic signaling pathways in the liver tissues, therefore holds great promise to develop potential preventive and therapeutic strategies for these patients.

## 7. Conclusions

This review summarizes the methodology of the major approaches used for the detection of pre-S gene deletions and pre-S deleted proteins, underscores the advantages and disadvantages of these approaches in practical operation, and highlights their applications in the prediction of a higher risk of HCC development and recurrence. The precise detection of pre-S gene deletions and pre-S deleted proteins via the proper use of these approaches is promising for the accurate identification and better management of high-risk patient populations, helping to improve their outcomes.

## Figures and Tables

**Table 2:** Summary of methodology, advantages, and disadvantages of four major approaches for the detection of HBV pre-S gene deletions and pre-S deleted proteins.

	Sanger DNA Sequencing-Based Approach	Pre-S Gene Chip-Based Approach	NGS-Based Approach	IHC Staining-Based Approach
**Sample Source**	Serum, plasma, or liver tissues	Serum, plasma, or liver tissues	Serum, plasma, or liver tissues	Liver tissues
**Sample Type**	DNA	DNA	DNA	Protein
**Detection Method**	Sanger DNA sequencing	Pre-S gene chip	NGS	IHC staining
**Detection Target**	Pre-S gene deletions	Pre-S gene deletions	Pre-S gene deletions	Pre-S deleted proteins
**Need of Agarose Gel Electrophoresis**	Yes	Yes	No	No
**Need of TA Cloning**	Yes	Yes	No	No
**Time of Each Assay**	2 to 4 days	1 to 3 days	1 to 2 days	2 days
**Cost of Each Assay**	USD~20 (for single PCR band)	USD~80 (for single PCR band)	USD~700	USD~30
**Advantages**	a. Easy to operate b. Provides sequence information	a. Easy to operate b. A little more time-efficient than the Sanger DNA sequencing-based approach	a. Much more efficient, sensitive, and accurate than the other approaches b. Provides sequence information c. Provides not only qualitative but also quantitative results	Provides information on the expression patterns of pre-S deleted proteins in the liver tissues
**Disadvantages**	a. Time-consuming b. Has limitations for analysis of the PCR bands with too-low intensities or too-close sizes c. Provides only qualitative or semiquantitative results	a. Has limitations for analysis of the PCR bands with too-low intensities or too-close sizes b. Cannot provide sequence information c. Provides only qualitative or semiquantitative results	a. Requires support from skilled instrument technicians and bioinformatics analysts b. More expensive than the other approaches	a. Provides results from partial but not whole liver tissues b. Has limitations for detection of proteins at too-low levels c. Provides only qualitative or semiquantitative results

Abbreviations: HBV, hepatitis B virus; NGS, next-generation sequencing; IHC, immunohistochemistry; TA, thymine–adenine; USD, United States dollar; PCR, polymerase chain reaction.
